# Obesity, Insulin Resistance, and Infertility in Women with Polyendocrine Metabolic Ovarian Syndrome: A Retrospective Cohort Study at a Tertiary Referral Medical Center in Qatar

**DOI:** 10.3390/healthcare14172677

**Published:** 2026-08-22

**Authors:** Tahani Ibrahim Alotoum, Husam Qush, Rafea Muftah AlGhanem, Ayman El-Menyar

**Affiliations:** 1 Department of Obstetrics and Gynecology, Military Medical City Hospital, Doha, 2352, Qatar; hu.qush@mmch.qa; 2 Department of Family Medicine, Military Medical City Hospital, Doha, 2352, Qatar; ralghanem@outlook.com; 3 Hamad Medical Corporation, Doha P.O. Box 3050, Qatar; 4 Weill Cornell Medicine, Doha P.O. Box 24144, Qatar

**Keywords:** Polyendocrine Metabolic Ovarian Syndrome, infertility, insulin resistance, obesity, hormonal profile, Polycystic Ovary Syndrome

## Abstract

**Background**: Polyendocrine Metabolic Ovarian Syndrome (PMOS), previously known as Polycystic Ovary Syndrome (PCOS), is one of the most common endocrine disorders affecting women of reproductive age and represents a major cause of infertility worldwide. It is associated with hormonal imbalance, ovulatory dysfunction, and metabolic disturbances, all of which can significantly impair reproductive outcomes and quality of life. We aimed to investigate the metabolic and hormonal markers in infertile women who had PMOS in one of the rapidly developing Middle Eastern countries. Methods: This was a retrospective observational cohort study conducted at the Military Medical Specialist Center in Qatar (2019–2024). Data were extracted from patient medical records, including demographic characteristics, clinical presentation, hormonal profiles, metabolic parameters, and details of fertility treatment. Women aged 18–40 years diagnosed with PMOS according to the Rotterdam criteria were included. Correlation coefficient analysis was performed to assess the associations between PMOS-related factors. Patients were categorized by BMI (normal, overweight, and obese). Results: The mean age of patients was 31.9 ± 5.3 years, and 43.8% of patients were obese. Primary infertility was more frequent than secondary infertility (61.8% vs. 38.2%). Women with secondary infertility were significantly older and had higher body mass index (BMI) (*p* = 0.001 and *p* = 0.01, respectively). Insulin resistance was prominent (mean Homeostatic Model Assessment for Insulin Resistance [HOMA-IR] of 4.04) and increased significantly with the increase in BMI (*p* = 0.01). BMI showed positive correlations with serum levels of glucose, insulin, HOMA-IR, and testosterone. Hormonal parameters were largely comparable between infertility groups, except for lower FSH levels in secondary infertility (*p* = 0.04). The proportion of PMOS based on the HOMA-IR category was 5.6% (HOMA-IR < 1.0), 23.4% (HOMA-IR 1–1.99), 20.2% (HOMA-IR 2–2.99), and 50.8% (HOMA-IR > 3.00). In PMOS patients, there was a significant association between obesity and HOMA-IR, with each 1-unit increase in HOMA-IR associated with a 14% increase in odds (crude odds ratio 1.14; 95% confidence interval 1.02–1.28, *p* = 0.02). Also, obesity was associated with low LH/FSH (crude odds ratio, 0.67; 95% CI, 0.45–0.99; *p* = 0.04). Results: The mean age of patients was 31.9 ± 5.3 years, and 43.8% of patients were obese. Primary infertility was more frequent than secondary infertility (61.8% vs. 38.2%). Insulin resistance was prominent (mean HOMA-IR 4.04) and increased significantly across BMI categories (3.0 in normal-weight vs. 4.8 in obese women, *p* = 0.01). BMI was positively correlated with HOMA-IR (r = 0.17, *p* = 0.02) and testosterone levels (r = 0.18, *p* = 0.01). Secondary infertility became more frequent with increasing BMI (*p* = 0.02). Each 1-unit increase in HOMA-IR was associated with a 14% increase in the odds of obesity (OR 1.14, 95% CI 1.02–1.28, *p* = 0.02). **Conclusions**: Among infertile women with PMOS, obesity and insulin resistance were prominent metabolic characteristics. These findings support routine screening for insulin resistance, particularly in overweight and obese women, together with weight management and individualized fertility treatment based on BMI and metabolic profiles.

## 1. Introduction

**Definition and epidemiology of PMOS:** Polyendocrine Metabolic Ovarian Syndrome (PMOS) is one of the most common endocrine disorders affecting women of reproductive age, with an estimated prevalence of 10–13% worldwide [1,2]. It is characterized by hyperandrogenism, ovulatory dysfunction, and polycystic ovarian morphology (PCOM), according to the Rotterdam diagnostic criteria [3,4,5,6,7]. Owing to its heterogeneous clinical presentation and variable diagnostic criteria, reported prevalence varies across different populations and regions.

**Transition from POCS to PMOS:** PMOS represents a terminology shift from PCOS [1,8,9,10,11]. Clinically, the transition from PCOS to PMOS has implications beyond nomenclature. This renaming supports comprehensive assessment in terms of insulin resistance, obesity, glucose metabolism, lipid profile, and cardiovascular risk, as well as menstrual, ovulatory, androgenic, and fertility-related features. In women with infertility and high burden of obesity and insulin resistance, the PMOS concept reinforces the use of integrated management that combines reproductive treatment, metabolic screening, lifestyle intervention, and individualized risk reduction [8,9,10,11]. It also addresses the importance of insulin resistance and obesity before or alongside ovulation induction or assisted reproduction. It better captures the phenotype seen in populations with high obesity and insulin resistance burden, including Gulf and Middle Eastern populations. It may support more region-specific research into metabolic phenotypes, cardiometabolic risk, infertility outcomes, and long-term follow-up.

**Clinical Features and Pathophysiology:** PMOS is a complex endocrine disorder characterized by hormonal imbalance and reproductive dysfunction. Clinical manifestations include menstrual irregularities, chronic anovulation, infertility, hirsutism, acne, androgenic alopecia, and polycystic ovarian morphology. These features result from disturbances in the hypothalamic–pituitary–ovarian axis, leading to alterations in luteinizing hormone (LH), follicle-stimulating hormone (FSH), androgen production, and ovarian follicular development [12,13].

**Metabolic Consequences and Obesity:** Beyond its reproductive manifestations, PMOS is increasingly recognized as a metabolic disorder. Insulin resistance represents one of its central pathophysiological mechanisms and is frequently associated with obesity, dyslipidemia, metabolic syndrome, and an increased risk of type 2 diabetes mellitus and cardiovascular disease. Homeostatic Model Assessment for Insulin Resistance (HOMA-IR) is widely used to evaluate metabolic dysfunction and has been associated with ovulatory dysfunction, infertility, and adverse reproductive outcomes in women with PMOS [14]. High HOMA-IR scores are often linked to ovulation failure, irregular periods, and difficulty conceiving. Lowering insulin resistance can help restore ovarian function [15,16,17].

PMOS demonstrates marked phenotypic heterogeneity, with as many as sixteen possible combinations of clinical and biochemical features described in the literature. Nevertheless, the Rotterdam consensus framework delineates four principal phenotypes derived from varying combinations of hyperandrogenism, ovulatory dysfunction, and polycystic ovarian morphology [17]. The Rotterdam Consensus Workshop redefined PMOS as a syndrome of ovarian dysfunction, with hyperandrogenism and polycystic ovarian morphology (PCOM) recognized as its principal diagnostic features [18]. Application of the 1990 National Institutes of Health (NIH) diagnostic criteria yields a global prevalence estimate of 6–8% for PMOS. On this basis, PMOS is widely regarded as the most common endocrine disorder affecting women of reproductive age and one of the most prevalent chronic conditions in this group [18].

**PMOS and Infertility in Qatar/Middle East:** The burden of PMOS appears to be particularly important in the Middle East, where obesity and insulin resistance are highly prevalent among women of reproductive age. These metabolic factors may contribute to infertility and adverse reproductive outcomes. Despite the increasing prevalence of PMOS and infertility in the region, data describing the clinical, hormonal, and metabolic characteristics of affected women in Qatar remain limited. Generating local evidence is therefore important to better understand disease patterns and optimize clinical management. Although some studies have investigated the clinical and metabolic features of PMOS in Middle Eastern populations, evidence from Qatar remains limited. Given the high burden of obesity and metabolic disorders in the region, region-specific data are needed to better characterize women with PMOS and infertility and to support evidence-based clinical management.

**Research Gap and Study Objective:** Given the heterogeneity of PMOS and variability in reported prevalence, this retrospective cohort study aims to (1) describe the clinical, metabolic, and hormonal characteristics of women with PMOS and infertility in Qatar; (2) compare these characteristics across BMI categories (normal weight, overweight, and obese); and (3) examine the associations between BMI, insulin resistance, hormonal parameters, and infertility type. This could help refine diagnostic approaches, clarify phenotypic patterns, and support the development of targeted management strategies.

## 2. Materials and Methods

This retrospective observational cohort study was conducted at the Military Medical Specialist Center, a tertiary referral center within the Qatar Armed Forces Medical Services (QAFMS), Doha, Qatar. The center provides specialized obstetrics and gynecology, including infertility services, primarily to military personnel, their dependents, and other eligible beneficiaries from across the State of Qatar. The study included women diagnosed with polycystic ovary syndrome (PMOS) and infertility who attended the center between January 2019 and December 2024. Data were retrospectively extracted from the electronic medical records during 2025 using a standardized data extraction form. Demographic characteristics, clinical presentation, hormonal profiles, metabolic parameters, and fertility treatment details were collected for analysis.

The study population included women aged 18–40 years with a confirmed diagnosis of PMOS based on the Rotterdam criteria [18,19], defined by the presence of at least two of the following three features after exclusion of other etiologies: (1) oligo- or anovulation, (2) clinical and/or biochemical hyperandrogenism, and (3) PCOM on ultrasound. The age range of 18–40 years was selected to include women of reproductive age, minimize the potential influence of perimenopausal hormonal changes on endocrine and metabolic parameters, and ensure a more homogeneous study population for evaluating PMOS-related infertility.

**Inclusion and exclusion criteria:** Eligible participants also had a documented history of infertility, defined as the inability to conceive after at least 12 months of regular, unprotected sexual intercourse. Patients were excluded if infertility was attributed to other causes, such as tubal factor infertility or male factor infertility, or if they had significant endocrine disorders, including hyperprolactinemia or hypothyroidism. According to the World Health Organization (WHO), primary infertility is defined as the inability to achieve a pregnancy despite at least 12 months of regular unprotected intercourse without any previous pregnancy, whereas secondary infertility refers to the inability to conceive following at least one previous pregnancy [20,21].

**Data collection** was performed using a standardized data extraction form developed for this study to ensure consistency. Collected variables included demographic data (age, body mass index, ethnicity, and lifestyle factors), medical and reproductive history (duration of infertility, age at PMOS diagnosis, and relevant family history), and menstrual characteristics (cycle regularity and symptoms of hyperandrogenism). Detailed hormonal and biochemical parameters were recorded, including fasting glucose, insulin levels, HOMA-IR, gonadotropins (LH and FSH), androgens (testosterone, DHEA-S, androstenedione), as well as thyroid-stimulating hormone and prolactin levels. HOMA-IR was calculated as Fasting Glucose in mmol/L × Fasting Insulin in μU/mL divided by 22.5 [22,23].

**Laboratory parameters**: All hormonal and biochemical measurements were performed in the hospital’s central laboratory using standardized routine clinical laboratory methods, including chemiluminescence. Calibration was performed following each reagent lot change or every 84 days when the same lot remained in use. Internal quality control was conducted at least once every 24 h, and results were accepted only when the control values were within the established laboratory limits. For descriptive purposes, HOMA-IR values were categorized as <1.0, 1.0–1.99, 2.0–2.99, and ≥3.0, with values ≥3.0 considered indicative of insulin resistance (Table 1).

**Ultrasound findings** were reviewed in terms of ovarian morphology, including ovarian volume, antral follicle count, and the presence of polycystic ovarian features.

The study was approved by the Institutional Review Board of the Qatar Armed Forces Medical Services (QAFMS) (Approval No. QAFMS/RRC/002/2026; approved on 03 May 2026). Given the retrospective nature of the study, the requirement for informed consent was waived. All data were anonymized prior to analysis.

**Statistical Analysis:** The distribution of continuous variables was assessed using the Shapiro–Wilk test and visual inspection of histograms and Q–Q plots (age and BMI were approximately normally distributed, whereas several biochemical and hormonal variables showed non-normal distributions). Normally distributed continuous variables were summarized as mean ± standard deviation, while non-normally distributed variables were presented as median and interquartile range. Categorical variables were summarized as frequencies and percentages. Comparisons between women with primary and secondary infertility were performed using the independent-samples t-test for normally distributed variables and the Mann–Whitney U test for non-normally distributed variables. Comparisons across normal-weight, overweight, and obese BMI categories were performed using one-way analysis of variance for normally distributed variables and the Kruskal–Wallis test for non-normally distributed variables. Categorical variables were compared using Pearson’s chi-square test or Fisher’s exact test when expected cell frequencies were small.

Associations between metabolic and hormonal parameters were evaluated using Spearman’s rank correlation coefficient because several variables were non-normally distributed. Univariable binary logistic regression analysis was performed to identify factors independently associated with obesity, defined as BMI ≥ 30 kg/m^2^. The model included age, infertility type, LH/FSH ratio, testosterone, and HOMA-IR, which were evaluated separately, and results were presented as crude odds ratios with 95% confidence intervals. All statistical tests were two-sided, and *p* < 0.05 was considered statistically significant. Statistical analyses were performed using [IBM SPSS Statistics for Windows, version 21.0; IBM Corp., Armonk, NY, USA].

## 3. Results

### 3.1. Baseline Characteristics

**Figure 1:**
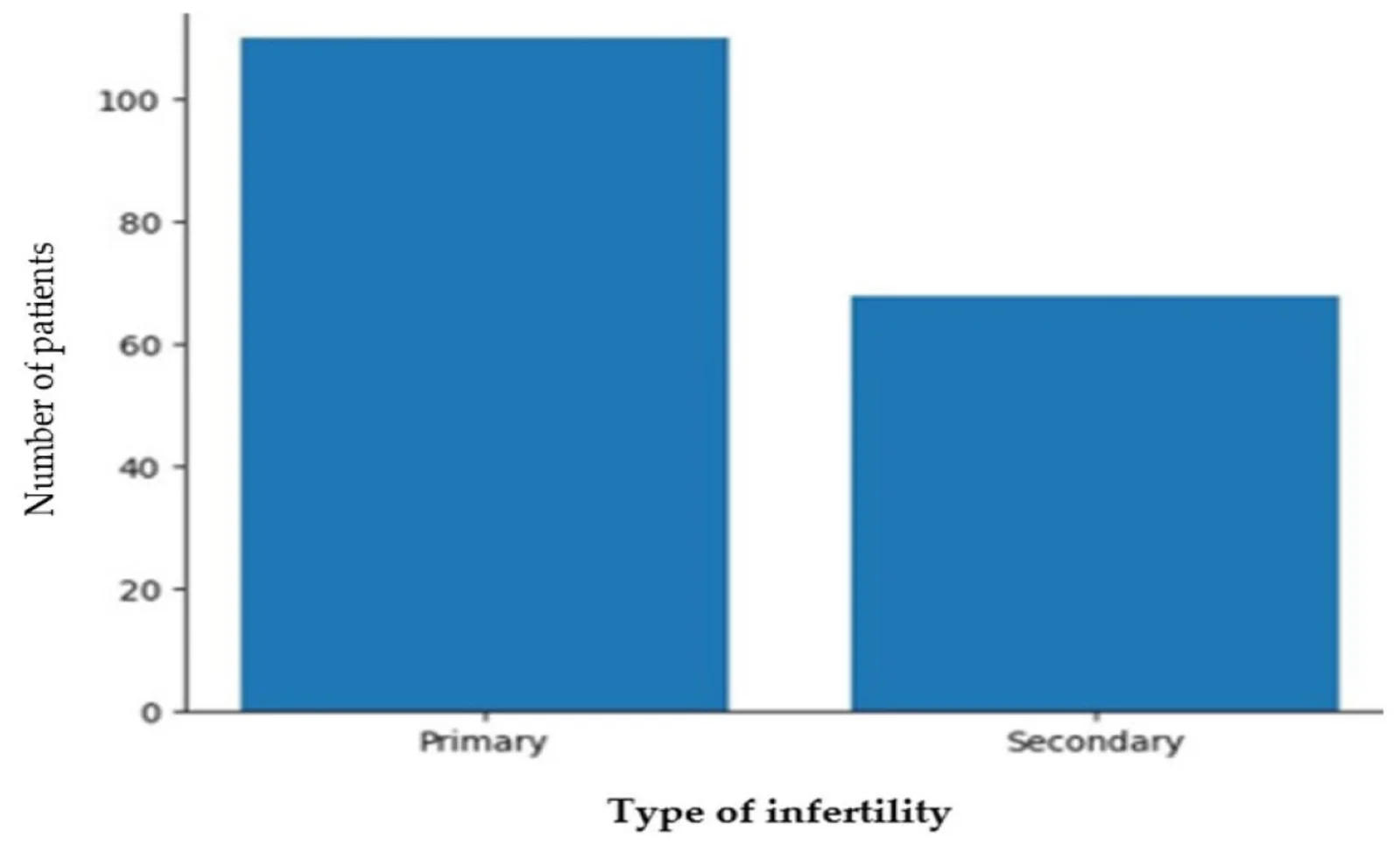
Distribution of infertility types among women with PMOS: Primary infertility was more prevalent in the study population, accounting for 61.8% of cases, compared to secondary infertility, which represented 38.2%. This finding highlights the association between PMOS and primary infertility in this cohort.

A total of 178 women with PMOS and infertility were included in the study. The mean age of the cohort was 31.9 ± 5.3 years, with approximately half of the patients belonging to the 31–40 years age group. Obesity was common, with 43.8% of patients classified as obese, while 24.2% had a normal body mass index (BMI). Primary infertility was more frequent (61.8%) compared to secondary infertility (38.2%). Most patients (70.8%) reported regular menstrual cycles, whereas the remaining had irregular cycles. (Table 2 and Figure 1).

### 3.2. Biochemical and Hormonal Profile

The biochemical profile demonstrated elevated fasting insulin levels (16.8 µU/mL) and a mean HOMA-IR of 4.04 ± 3.33, indicating insulin resistance within the cohort. The mean LH/FSH ratio was 1.5, and androgen levels were elevated, with mean testosterone of 116.2 ng/dL and DHEA-S of 207.6 µg/dL (Table 3).

### 3.3. Comparison by Infertility Type

Women with secondary infertility were significantly older than those with primary infertility (33.8 vs. 30.8 years, *p* = 0.001) and had a higher BMI (30.6 vs. 28.3 kg/m^2^, *p* = 0.01) (Table 4). There was no significant difference in menstrual regularity between the groups. Hormonal parameters were comparable between the two groups. However, women with secondary infertility had significantly lower FSH levels than those with primary infertility (5.8 ± 1.5 vs. 6.6 ± 2.9 mIU/L, *p* = 0.04).

### 3.4. Body Mass Index

Almost 44% of PMOS were obese. Obesity was associated with worsened metabolic parameters. HOMA-IR increased progressively from normal-weight individuals to obese patients (3.0 vs. 4.8, *p* = 0.01). Fasting glucose increased significantly across BMI categories (*p* = 0.02), whereas fasting insulin showed an increasing trend that did not reach statistical significance (*p* = 0.06).

**Figure 2:**
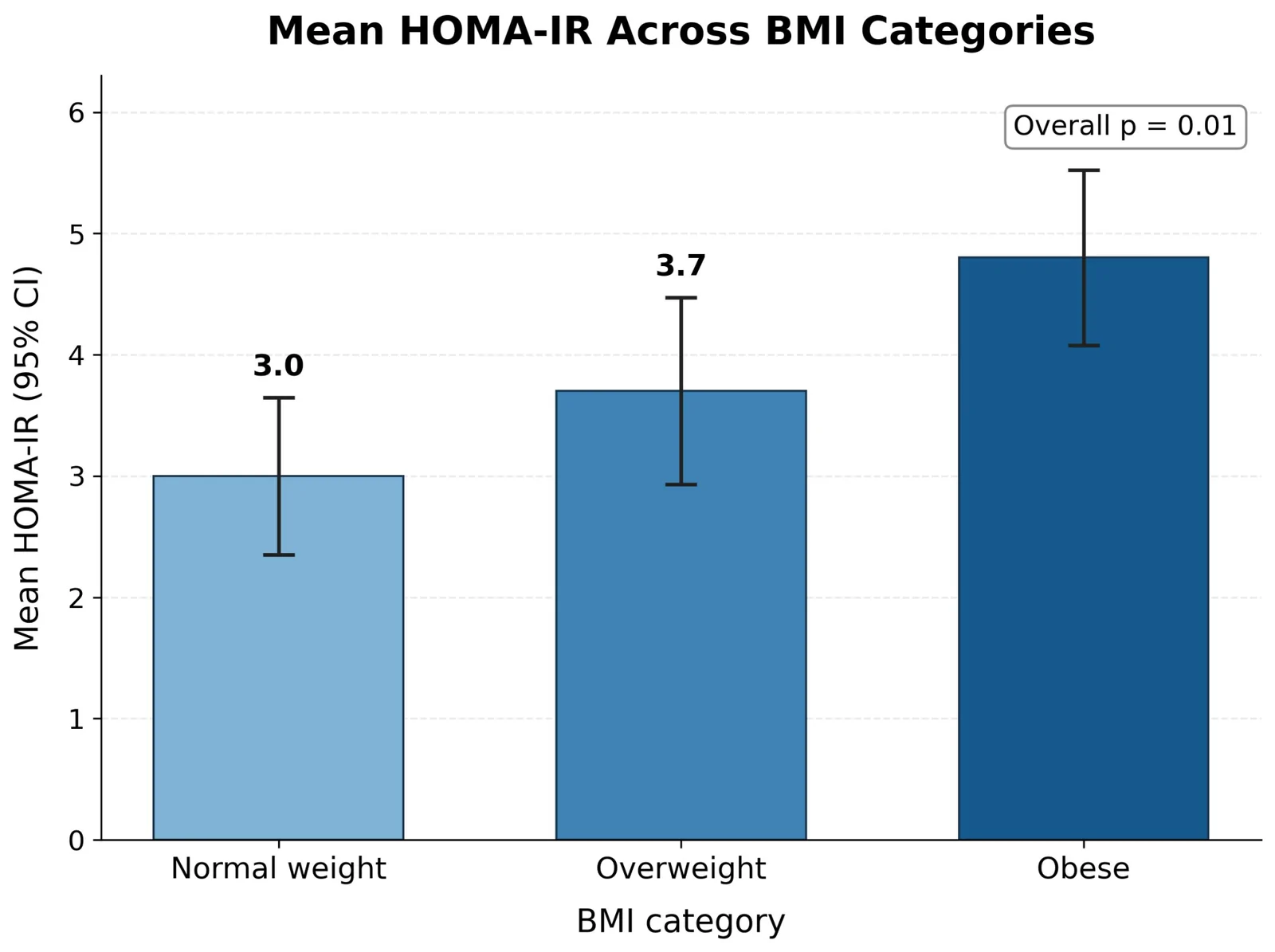
Trend of insulin resistance (HOMA-IR) across BMI categories. HOMA-IR values increased progressively across BMI categories, with the highest levels observed in obese patients. This trend indicates a significant relationship between increasing adiposity and worsening insulin resistance. These findings support the role of obesity as a key contributor to metabolic dysfunction in women with PMOS.

The type of infertility differed significantly across BMI categories, with primary infertility more common in normal-weight women, whereas secondary infertility was more frequent among obese women (*p* = 0.02). Hormonal markers did not differ significantly across BMI groups, although androgens showed a non-significant increasing trend. (Table 5 and Figure 2).

### 3.5. Correlation Analysis

Spearman correlation analysis demonstrated that age had a modest positive correlation with fasting glucose (r = 0.20) and negative correlations with LH, testosterone, and LH/FSH ratio. BMI showed significant positive correlations with glucose, insulin, HOMA-IR, and testosterone. Positive correlations were observed between HOMA-IR and insulin (r = 0.92) and between HOMA-IR and glucose (r = 0.41). Testosterone levels were positively correlated with BMI, LH, DHEA-S, and the LH/FSH ratio (Table 6).

### 3.6. Logistic Regression Analysis 

Univariable binary logistic regression analysis demonstrated that higher HOMA-IR was significantly associated with obesity, with each one-unit increase associated with a 14% increase in the odds of obesity (crude OR 1.14; 95% CI 1.02–1.28; *p* = 0.02). Lower LH/FSH ratio was also significantly associated with obesity (crude OR 0.67; 95% CI 0.45–0.99; *p* = 0.04).

**Figure 3:**
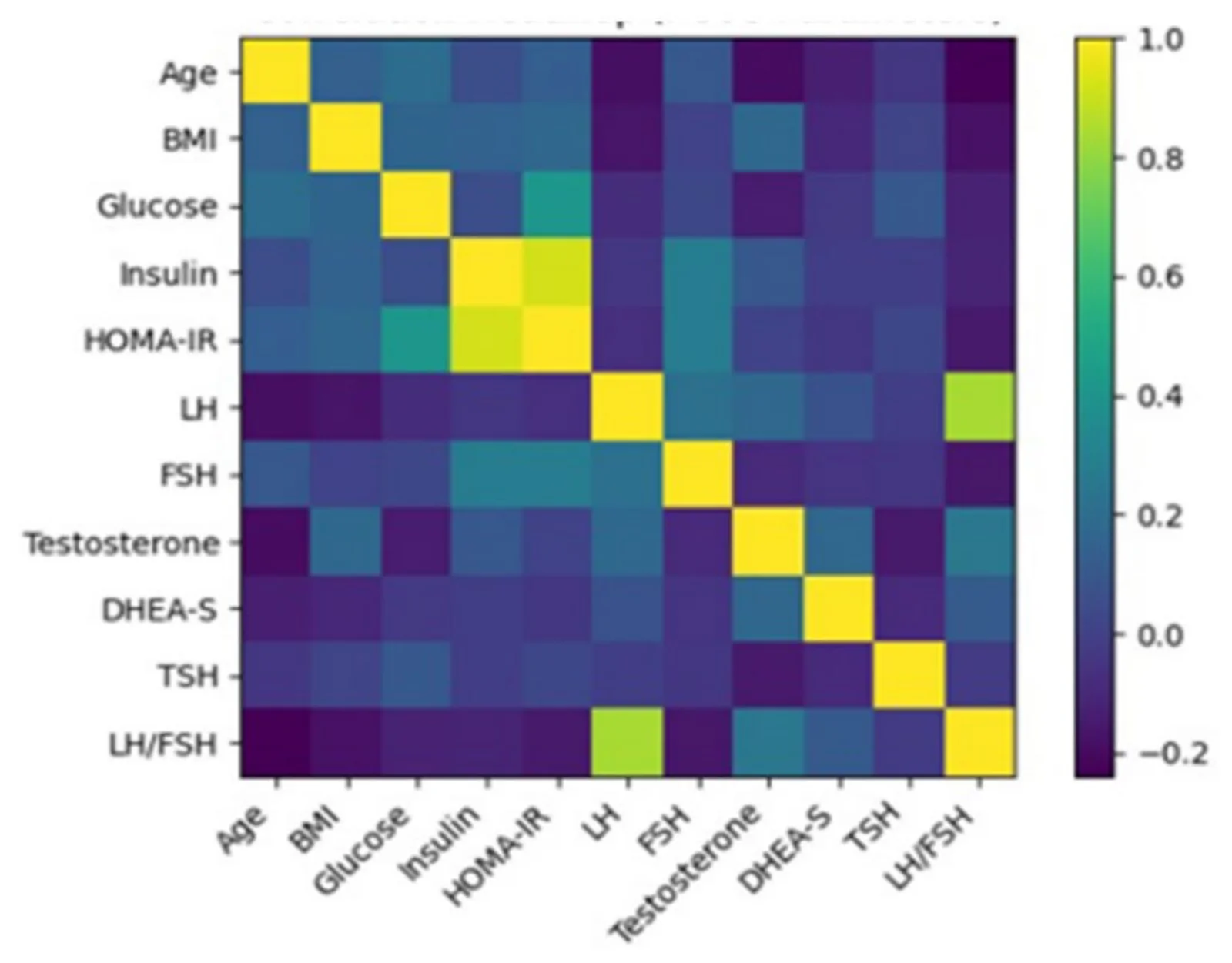
The correlation heatmap for PMOS parameters.

Figure 3 shows the correlation heatmap. This figure shows the correlation heatmap demonstrates BMI correlated positively with insulin resistance and testosterone, while strong associations between insulin and HOMA-IR validate metabolic dysfunction. Hormonal correlations highlight the interplay between androgen excess and gonadotropin imbalance in PMOS. The proportion of PMOS based on the HOMA-IR category was 5.6% (HOMA-IR < 1.0), 23.4% (HOMA-IR 1–1.99), 20.2% (HOMA-IR 2–2.99) and 50.8% (HOMA-IR > 3.00) (Table 7).

## 4. Discussion

This study provides a comprehensive evaluation of the clinical, metabolic, and reproductive characteristics of women with PMOS in a regional cohort stratified by BMI. The findings highlight the substantial burden of PMOS as a multifaceted endocrine disorder, extending beyond reproductive dysfunction to include significant metabolic derangements. The high prevalence of obesity, insulin resistance, and hyperandrogenism observed in this cohort reinforces the importance of early identification and integrated management of PMOS, particularly in populations at higher metabolic risk.

Overall, our findings suggest that the metabolic phenotype of women with PMOS in Qatar is characterized by a close interplay between obesity, insulin resistance, and reproductive dysfunction rather than isolated hormonal abnormalities. This pattern is clinically important because it supports the concept that metabolic disturbances should be considered alongside reproductive manifestations when evaluating women with PMOS. These observations also provide region-specific evidence that may help inform clinical practice and future research in Gulf populations with a high burden of obesity and metabolic disease.

In our cohort, the predominance of primary infertility (61.8%) compared to secondary infertility (38.2%) underscores the significant reproductive impact of PMOS, consistent with its well-established role as the leading cause of anovulatory infertility, accounting for nearly 70–80% of cases [24]. The mean age (31.9 ± 5.3 years) and the high prevalence of obesity (43.8%) reflect a demographic pattern commonly reported in regional studies, in which delayed presentation and increasing metabolic risk factors contribute to the disease burden.

When compared with regional data, our findings are consistent with studies from the Gulf Cooperation Council (GCC) countries, where PMOS prevalence among infertile women ranges from 21.5% to 35.5%, with even higher variability reported in Saudi Arabia (18–56%) [1,2,3,4,5,6,7]. This regional pattern aligns with global evidence suggesting that Middle Eastern populations exhibit a disproportionately higher burden of PMOS, with prevalence estimates reaching up to 16% in large meta-analyses [25]. These similarities support the notion that genetic predisposition, combined with environmental and lifestyle factors such as sedentary behavior and dietary changes, plays a significant role in shaping the epidemiology of PMOS in this region [26,27,28] (Table 8).

Furthermore, the regional comparisons presented in Table 8 and Table 9 indicate that the metabolic and reproductive profile observed in the present Qatari cohort closely resembles reports from neighboring Gulf countries, particularly with respect to the high prevalence of obesity, insulin resistance, and primary infertility. These similarities may reflect shared demographic characteristics, lifestyle patterns, and the high burden of metabolic disorders across the Gulf region. In contrast, studies from East Asia and Western populations generally report lower obesity rates or different phenotypic patterns, emphasizing the importance of interpreting PMOS characteristics within their regional context. Although the overall metabolic phenotype was broadly comparable to that reported in neighboring Gulf countries, the present study provides one of the few detailed Qatari cohorts integrating hormonal, metabolic, and infertility characteristics within the same population, thereby addressing an important regional evidence gap. Table 8 shows a comparison of PMOS characteristics. Qatar, Saudi Arabia (KSA) and four GCC countries (Qatar, Kuwait, Oman, KSA) demonstrate higher obesity and insulin resistance compared to global cohorts, with a stronger association with primary infertility, reflecting a more severe metabolic phenotype (Qatar and KSA are neighbor GCC countries [25,27,28].

From a metabolic perspective, elevated HOMA-IR values and fasting insulin levels observed in this cohort confirm the central role of insulin resistance in PMOS pathophysiology. These findings are in line with prior studies demonstrating that insulin resistance is present in up to 95% of obese and 75% of lean women with PMOS [17,18,22]. More than half of the PMOS cohort had HOMA-IR of >3.0 in the present study. Furthermore, the observed correlation between BMI and metabolic indices highlights the contribution of visceral adiposity to cardiometabolic risk, as previously reported [15,29]. Obese patients with PMOS are more prone to insulin resistance (61.5%) and metabolic syndrome (69.2%) [23]. Moreover, PMOS itself may act as an intrinsic risk for metabolic abnormalities. However, females with PMOS may have metabolic disturbances regardless of their BMI [23]. Excess adiposity may contribute to more pronounced metabolic and endocrine abnormalities in women with PMOS. Although obesity was associated with greater insulin resistance and metabolic abnormalities, the presence of PMOS across different BMI categories indicates that obesity alone does not explain the pathogenesis of the syndrome. Instead, obesity appears to exacerbate the metabolic and reproductive manifestations of an underlying endocrine disorder.

Hormonal analysis in our cohort demonstrated elevated LH/FSH ratios and increased androgen levels, consistent with the classical endocrine profile of PMOS [30,31,32,33]. These findings further support the role of disrupted gonadotropin secretion and androgen excess in impairing folliculogenesis and ovulation. Non-obese PMOS is associated with a higher LH/FSH ratio; however, obese women with PMOS tend to have lower LH levels and a lower LH/FSH ratio compared to their non-obese PMOS counterparts. Excess peripheral fat in obese PMOS drives insulin resistance and estrogen overproduction, which chronically suppresses the typical LH surge [34,35].

This finding suggests that lean and obese PMOS may represent overlapping but biologically distinct phenotypes. While lean PMOS appears to be characterized predominantly by hypothalamic–pituitary–ovarian axis dysfunction, obesity introduces additional metabolic disturbances that may attenuate the relative increase in LH secretion without eliminating the underlying endocrine abnormality.

Although obesity was strongly associated with insulin resistance and adverse metabolic parameters, most hormonal markers did not differ significantly across BMI categories. One possible explanation is that the endocrine abnormalities characteristic of PMOS are intrinsic to the syndrome itself and may already be present regardless of body weight. In contrast, obesity appears to exert a greater effect on metabolic dysfunction than on circulating reproductive hormone concentrations. Furthermore, the substantial inter-individual variability in hormonal measurements and the heterogeneous phenotypes of PMOS may have reduced the ability to detect significant between-group differences. These findings suggest that obesity primarily modifies the metabolic expression of PMOS rather than uniformly altering its hormonal profile.

In this study, there was a lack of significant metabolic difference between primary and secondary infertility, despite differences in age and BMI. This observation contrasts with previous reports that suggested distinct metabolic phenotypes between infertility types [28]. This discrepancy may reflect population-specific factors or differences in study design and sample characteristics, highlighting the need for further research to clarify these associations.

One possible explanation is that insulin resistance and metabolic dysfunction are core features of PMOS regardless of infertility subtype. Therefore, once women meet the diagnostic criteria for PMOS, the metabolic burden may be more strongly influenced by the syndrome itself and BMI than by whether infertility is primary or secondary. This interpretation should be confirmed in larger prospective studies.

Additionally, our study provides further insight into the relationship between obesity and infertility patterns. While primary infertility was more common overall, secondary infertility was more prevalent among obese women, suggesting a potential shift in reproductive phenotype with increasing BMI. This finding partially agrees with existing literature but also indicates a more complex interaction between metabolic and reproductive factors than previously described [36,37,38,39].

Importantly, this study contributes to the existing literature by providing integrated clinical, hormonal, and metabolic data from a single cohort, allowing for a comprehensive evaluation of PMOS in a real-world setting. Unlike some previous studies that focused on isolated parameters, our analysis highlights the interplay between endocrine dysfunction, metabolic abnormalities, and reproductive outcomes, thereby offering a more holistic understanding of the syndrome. Table 9 summarizes the prevalence and risk factors by region [6,19,28,40]. Taken together, these regional comparisons strengthen the external validity of our findings by demonstrating that the clinical and metabolic characteristics observed in Qatar are largely consistent with those reported across the Gulf region while highlighting important geographic differences in PMOS phenotype worldwide.

**Table 9:** Regional comparison of incidence/prevalence and risk factors of PMOS.

Country	Incidence/Prevalence of PCOS	Risk Factors
Middle East [28]	High prevalence: Pooled prevalence is 11.9% using Rotterdam criteria, reaching as high as 18.8% in Gulf Arab states.	Highest rates of hirsutism globally. Significant genetic admixture. High infertility rates (2.4% to 3.7%).
China [19]	Fastest growth: 5.3 million cases in 2021 (15% of global burden). Projected to increase 27% by 2036	Rapid lifestyle westernization is a major driver. “Lean PMOS” is common, with metabolic risks present at lower BMIs.
Japan [40]	Age-standardized incidence rate of 129.5 per 100,000	Distinct genetic predisposition with lower obesity rates but significant metabolic syndrome risk.
South Asia [40]	Highest raw case count in South Asia (269.8 per 100,000 prevalence).	High incidence of insulin resistance and metabolic syndrome, consanguinity, central obesity.
New Zealand [6]	Age-standardized incidence rate of 114.6 per 100,000	Western lifestyle patterns (diet, physical activity). High healthcare access leads to better detection rates.
Italy [6]	Highest prevalence: Age-standardized prevalence rate of 8113.16 per 100,000	Genetic background (Mediterranean) may influence the manifestation of certain features, such as higher rates of hirsutism
USA [6]	High prevalence: Ranked among top tier nations for PMOS with an age-standardized prevalence rate of 1958.7 per 100,000	High obesity rates, particularly in certain ethnic groups.

From a clinical perspective, these findings emphasize the importance of routine screening for insulin resistance using appropriate metabolic assessment in women with PMOS presenting with infertility, particularly those with obesity, to facilitate early risk stratification and individualized management. Lifestyle modification, including weight management and regular physical activity, should remain the cornerstone of first-line therapy. In addition, fertility treatment strategies should be tailored according to each patient’s BMI and metabolic profile to optimize both reproductive and metabolic outcomes. These findings may also help inform clinical decision-making and future care pathways in similar healthcare settings. These findings provide region-specific evidence that supports routine metabolic screening and individualized fertility management in women with PMOS in Qatar and may assist clinicians in prioritizing early interventions for obesity and insulin resistance.

### 4.1. Study Limitations

This study has several limitations. First, its retrospective design may have introduced information bias because the analyses relied on data recorded in electronic medical records. Second, as a single-center study, the findings may not be fully generalizable to other populations or healthcare settings. Third, although the sample size was adequate for the primary analyses, it may have limited the statistical power to detect smaller associations in some subgroup analyses. Residual confounding from unmeasured factors, including lifestyle characteristics, dietary habits, physical activity, and medication use, cannot be excluded. Nevertheless, this study provides valuable real-world data on women with PMOS and infertility in Qatar and offers important region-specific evidence to guide future prospective multicenter studies. No formal correction for multiple testing was applied because the correlation and secondary analyses were exploratory.

### 4.2. Recommendations

Management strategies should adopt a multidisciplinary approach that addresses both reproductive and metabolic aspects of the disease. Specifically, management may include structured lifestyle modification, weight reduction, nutritional counseling, optimization of insulin resistance when clinically indicated, and coordinated care involving gynecologists, endocrinologists, dietitians, and fertility specialists. Lifestyle modification, including weight management and physical activity, should be considered a cornerstone of treatment. Furthermore, individualized treatment plans should be developed according to patient phenotype, as variability in hormonal and metabolic profiles may influence treatment response and reproductive outcomes. Finally, further large-scale prospective studies are recommended to better elucidate the causal relationships between metabolic dysfunction and infertility in PMOS and to explore targeted therapeutic interventions.

Future prospective studies are needed to clarify the causal relationships between obesity, insulin resistance, and reproductive dysfunction in women with PMOS. In addition, interventional studies evaluating structured weight loss programs, lifestyle modification, and insulin-sensitizing therapies such as metformin are warranted to determine their effects on both reproductive and metabolic outcomes. Future research should also investigate treatment responses according to baseline metabolic phenotype, including BMI and HOMA-IR, to support more personalized management strategies. This study adds region-specific, real-world evidence from Qatar demonstrating the close association of obesity and insulin resistance with reproductive dysfunction among infertile women with PMOS, an understudied population in the existing literature. Such an approach may improve risk stratification and help individualize fertility treatment strategies, based on BMI and metabolic profile, in this population. Future prospective studies are needed to assess causality and confirm these observed associations, while interventional studies should evaluate the effects of weight loss programs, lifestyle modification, and metformin therapy, as well as treatment outcomes according to patients’ metabolic profiles.

## 5. Conclusions

This study demonstrates a significant association between PMOS, obesity, insulin resistance, and infertility. While metabolic disturbances play a central role, infertility in PMOS appears to result from a complex interaction between endocrine and metabolic factors. Obesity emerges as an important modifiable risk factor that exacerbates both metabolic and reproductive dysfunction. These findings support the routine assessment of insulin resistance in women with PMOS presenting with infertility, particularly those with obesity, and highlight the importance of integrating metabolic optimization, including weight management and lifestyle modification, as first-line interventions into fertility management.

## Figures and Tables

**Table 1:** Laboratory methods, parameters and normal values.

Parameter	Assay/Method	Platform	Analytical Sensitivity	Analytical Specificity
Glucose	Glucose HK Gen.3 (GLUC3), enzymatic hexokinase method	cobas c 503	LoD = 0.11 mmol/L (2 mg/dL) LoQ = 0.11 mmol/L	No clinically defined % specificity; assay specificity evaluated by interference studies.
Insulin	Elecsys Insulin, ECLIA	cobas e 801	LoD = 0.4 μIU/mL (2.78 pmol/L) LoQ = 1.0 μIU/mL (6.95 pmol/L)	Cross-reactivity: bovine insulin 9.2%, porcine insulin 22.2%, human proinsulin 0.36%; C-peptide, glucagon, somatostatin and IGF-I not detectable.
LH	Elecsys LH, ECLIA	cobas e 801	LoD = 0.3 mIU/mL LoQ = 1.0 mIU/mL	FSH 0.005%, hCG 0.003%; TSH, hGH and hPL not detectable.
FSH	Elecsys FSH, ECLIA	cobas e 801	LoD = 0.3 mIU/mL LoQ = 1.0 mIU/mL	LH 0.022%, hCG 0.004%; TSH, hGH and hPL not detectable.
TSH	Elecsys TSH, ECLIA	cobas e 801	LoD = 0.005 μIU/mL LoQ = 0.005 μIU/mL	LH and FSH 0%; hGH and hCG not detectable.
Testosterone	Elecsys Testosterone II, ECLIA	cobas e 801	LoD = 2.50 ng/dL (0.087 nmol/L) LoQ = 12.0 ng/dL (0.416 nmol/L)	Cross-reactivity/interference is analyte-specific; the assay is standardized to ID-GC/MS. Nandrolone can interfere and is specifically contraindicated in the method sheet.
DHEA-S	Elecsys DHEA-S, ECLIA	cobas e 801	LoD = 0.2 μg/dL (0.005 μmol/L) LoQ = 3 μg/dL (0.081 μmol/L)	Cross-reactivity: androstenedione 10.8%, DHEA 8.9%, androsterone 2.1%, testosterone 2.55%, progesterone 1.32%; estradiol/estriol not detectable.
Laboratory parameter			Normal reference range	
Fasting glucose			3.3–5.5 mmol/L	
Insulin			2.6–24.9 μU/mL	
FSH–Follicular phase			3–12 IU/L	
FSH–Ovulation phase			4–22 IU/L	
FSH–Luteal phase			2–8 IU/L	
FSH–Postmenopausal phase			26–135 IU/L	
LH–Follicular phase			2–13 IU/L	
LH–Ovulation phase			14–96 IU/L	
LH–Luteal phase			1–11 IU/L	
LH–Postmenopausal phase			8–59 IU/L	
Total testosterone			0.29–1.67 nmol/L	
TSH			0.3–4.2 mIU/L	
DHEA-S			1.650–9.150 μmol/L	

**Table 2:** Descriptive analysis for PMOS and infertility (*n* = 178).

Variables	Value
**Age** (mean ± SD) years	31.9 ± 5.3
20–30 years	76 (42.7%)
31–40 years	89 (50.0%)
≥41 years	13 (7.3%)
**Body mass index**	29.5 ± 6.3
Normal	43 (24.2%)
Overweight	57 (32.0%)
Obese	78 (43.8%)
**Type of infertility**	
Primary	110 (61.8%)
Secondary	68 (38.2%)
**Menstrual Cycle Regularity**	
Regular	126 (70.8%)
Irregular	52 (29.2%)

Values are presented as mean ± standard deviation (SD) or frequency (%).

**Table 3:** Biochemical and hormonal parameters in PMOS patients.

Variables	Value
**Fasting Glucose (mmol/L)**	5.3 ± 1.5
**Fasting Insulin (2–20 µU/mL)**	16.8 ± 12.7
**HOMA-IR**	4.04 ± 3.33
**LH (IU/L)**	8.9 ± 6.4
**FSH (mIU/L)**	6.3 ± 2.5
**LH/FSH ratio**	1.5 ± 0.9
**Testosterone (ng/dL)**	116.2 ± 69.2
**DHEA-S (µg/dL)**	207.6 ± 98.6
**TSH (mIU/L)**	3.2 ± 1.9

All values are expressed as mean ± SD.

**Table 4:** Comparison of clinical and hormonal parameters in PMOS patients with primary and secondary infertility.

Variables	Primary Infertility (n = 110)	Secondary Infertility (n = 68)	*p*-Value
**Age** (mean ± SD) years	30.8 ± 5.1	33.8 ± 5.4	0.001
**BMI** (kg/m^2^), (mean ± SD)	28.3 ± 5.9	30.6 ± 5.7	0.01
**Menstrual Cycle Regularity**			
Regular	82 (74.5%)	44 (64.7%)	0.17
Irregular	28 (25.5%)	24 (35.3%)	0.17
**HOMA-IR**	4.1 ± 3.5	3.9 ± 2.9	0.86
**Fasting Glucose (mmol/L)**	5.4 ± 1.6	5.2 ± 1.0	0.47
**Fasting Insulin (µU/mL)**	16.9 ± 13.8	16.6 ± 11.0	0.86
**LH (IU/L)**	9.6 ± 7.5	7.8 ± 3.9	0.07
**FSH (mIU/L)**	6.6 ± 2.9	5.8 ± 1.5	0.04
**LH/FSH ratio**	1.5 ± 0.9	1.4 ± 0.8	0.42
**Testosterone (ng/dL)**	121.9 ± 76.3	106.9 ± 55.1	0.16
**DHEA-S (µg/dL)**	212.5 ± 94.5	199.7 ± 105.3	0.40
**TSH (mIU/L)**	3.3 ± 1.8	3.1 ± 2.3	0.64

Continuous variables are shown as mean ± SD; categorical variables as n (%).

**Table 5:** Comparison of clinical and hormonal parameters in normal, overweight and obese patients with PMOS.

Variables	Normal (BMI < 25 kg/m^2^) (n = 43)	Overweight (BMI 25–30 kg/m^2^) (n = 57)	Obese (BMI > 30 kg/m^2^) (n = 78)	*p* Value
**Age** (mean ± SD) years	30.3 ± 5.1	32.3 ± 5.2	32.6 ± 5.5	0.06
**BMI** (kg/m^2^), (mean ± SD)	21.7 ± 2.6	27.5 ± 1.3	34.6 ± 3.8	0.001
**Type of infertility**				
Primary	32 (74.4%)	38 (66.7%)	40 (51.3%)	0.02
Secondary	11 (25.6%)	19 (33.3%)	38 (48.7%)	0.02
**Menstrual Cycle Regularity**				
Regular	30 (69.8%)	40 (70.2%)	56 (71.8%)	0.96
Irregular	13 (30.2%)	17 (29.8%)	22 (28.2%)	0.96
**HOMA-IR**	3.0 ± 2.1	3.7 ± 2.9	4.8 ± 3.2	0.01
**Fasting Glucose (mmol/L)**	4.9 ± 0.4	5.2 ± 0.6	5.7 ± 2.1	0.02
**Fasting Insulin (µU/mL)**	14.1 ± 16.6	15.6 ± 10.7	19.3 ± 11.5	0.06
**LH (IU/L)**	9.9 ± 7.5	9.5 ± 7.8	7.9 ± 4.1	0.18
**FSH (mIU/L)**	6.3 ± 1.9	6.1 ± 1.4	6.5 ± 3.4	0.64
**LH/FSH ratio**	1.7 ± 1.2	1.5 ± 0.9	1.3 ± 0.7	0.09
**Testosterone (ng/dL)**	101.9 ± 48.7	109.6 ± 62.4	116.9 ± 69.2	0.08
**DHEA-S (µg/dL)**	214.9 ± 97.8	212.6 ± 92.7	199.9 ± 103.8	0.65
**TSH (mIU/L)**	3.6 ± 1.9	2.9 ± 1.6	3.1 ± 2.2	0.25

Continuous variables are expressed as mean ± SD. *p*-values represent overall differences across BMI groups.

**Table 6:** Spearman’s correlation for metabolic and hormonal parameters in the study population.

Variable	Statistics	Age	BMI	Glucose	Insulin	HOMA-IR	LHIUL	FSH	Testosterone	DHEA-S	TSH	LH/FSH
**Age**	Correlation	-	ρ = 0.14	ρ = 0.20 **	ρ = 0.06	ρ = 0.13	ρ = 0.19 **	ρ = 0.10	ρ = 0.204 **	ρ = −0.136	ρ = −0.041	ρ = −0.24 **
	*p* value	-	*p* = 0.05	***p*** ** = 0.005**	*p* = 0.38	*p* = 0.08	***p*** ** = 0.008**	*p* = 0.17	***p*** ** = 0.006**	*p* = 0.070	*p* = 0.591	***p*** ** = 0.001**
**BMI**	Correlation	ρ = 0.14	-	ρ = 0.16 *	ρ = 0.15 *	ρ = 0.17 *	ρ = 0.17 *	ρ = 0.007	ρ = 0.18 *	ρ = −0.10	ρ = 0.02	ρ = −0.18 *
	*p* value	*p* = 0.05		***p*** ** = 0.02**	***p*** ** = 0.03**	***p*** ** = 0.02**	***p*** ** = 0.01**	*p* = 0.92	***p*** ** = 0.01**	*p* = 0.18	*p* = 0.73	***p*** ** = 0.01**
**Glucose**	Correlation	ρ = 0.20 **	ρ = 0.16 *	-	ρ = 0.06	ρ = 0.41 **	ρ = −0.08	ρ = 0.03	ρ = −0.14	ρ = −0.03	ρ = 0.10	ρ = −0.12
	*p* value	***p*** ** = 0.005**	***p*** ** = 0.02**		*p* = 0.38	***p*** ** = 0.001**	*p* = 0.28	*p* = 0.67	*p* = 0.05	*p* = 0.60	*p* = 0.15	*p* = 0.10
**Insulin**	Correlation	ρ = 0.06	ρ = 0.15 *	ρ = 0.06	-	ρ = 0.92 **	ρ = −0.04	ρ = 0.28 **	ρ = 0.10	ρ = −0.01	ρ = −0.01	ρ = −0.11
	*p* value	*p* = 0.38	***p*** ** = 0.03**	*p* = 0.38		***p*** ** = 0.001**	*p* = 0.51	***p*** ** = 0.001**	*p* = 0.14	*p* = 0.81	*p* = 0.87	*p* = 0.13
**HOMA-IR**	Correlation	ρ = 0.13	ρ = 0.17 *	ρ = 0.41 **	ρ = 0.92 **	-	ρ = −0.07	ρ = 0.28 **	ρ = 0.01	ρ = −0.04	ρ = 0.03	ρ = −0.15 *
	*p* value	*p* = 0.08	***p*** ** = 0.02**	***p*** ** = 0.001**	***p*** ** = 0.001**		*p* = 0.33	***p*** ** = 0.001**	*p* = 0.81	*p* = 0.57	*p* = 0.67	***p*** ** = 0.04**
**LH**	Correlation	Ρ = 0.19 **	ρ = 0.17 *	ρ = −0.08	ρ = −0.04	ρ = −0.07	-	ρ = 0.22 **	ρ = 0.18 *	ρ = 0.07	ρ = −0.01	ρ = 0.84 **
	*p* value	***p*** ** = 0.008**	***p*** ** = 0.01**	*p* = 0.28	*p* = 0.51	*p* = 0.33		***p*** ** = 0.003**	***p*** ** = 0.01**	*p* = 0.34	*p* = 0.80	***p*** ** = 0.001**
**FSH**	Correlation	ρ = 0.10	ρ = 0.007	ρ = 0.03	ρ = 0.28 **	ρ = 0.28 **	ρ = 0.22 **	-	ρ = −0.09	ρ = −0.05	ρ = −0.04	ρ = −0.16 *
	*p* value	*p* = 0.17	*p* = 0.92	*p* = 0.67	***p*** ** = 0.001**	***p*** ** = 0.001**	***p*** ** = 0.003**		*p* = 0.19	*p* = 0.48	*p* = 0.56	***p*** ** = 0.02**
**Testosterone**	Correlation	ρ = −0.20 **	ρ = 0.18 *	ρ = −0.14	ρ = 0.10	ρ = 0.01	ρ = 0.18 *	ρ = −0.09	-	ρ = 0.17 *	ρ = −0.15 *	ρ = 0.25 **
	*p* value	***p*** ** = 0.006**	***p*** ** = 0.01**	*p* = 0.05	*p* = 0.14	*p* = 0.81	***p*** ** = 0.01**	*p* = 0.19		***p*** ** = 0.01**	*p* = 0.03	***p*** ** = 0.001**
**DHEA-S**	Correlation	ρ = −0.13	ρ = −0.10	ρ = −0.03	ρ = −0.01	ρ = −0.04	ρ = 0.07	ρ = −0.05	ρ = 0.17 *	-	ρ = −0.09	ρ = 0.11
	*p* value	*p* = 0.07	*p* = 0.18	*p* = 0.60	*p* = 0.81	*p* = 0.57	*p* = 0.34	*p* = 0.48	***p*** ** = 0.01**		*p* = 0.22	*p* = 0.11
**TSH**	Correlation	ρ = −0.04	ρ = 0.02	ρ = 0.10	ρ = −0.01	ρ = 0.03	ρ = −0.01	ρ = −0.04	ρ = −0.15 *	ρ = −0.09	-	ρ = −0.02
	*p* value	*p* = 0.59	*p* = 0.73	*p* = 0.15	*p* = 0.87	*p* = 0.67	*p* = 0.80	*p* = 0.56	***p*** ** = 0.03**	*p* = 0.22		*p* = 0.79
**LH/FSH**	Correlation	ρ = −0.24 **	ρ = 0.18 *	ρ = −0.12	ρ = −0.11	ρ = −0.15 *	ρ = 0.84 **	ρ = 0.16 *	ρ = 0.25 **	ρ = 0.11	ρ = −0.02	-
	*p* value	***p*** ** = 0.001**	***p*** ** = 0.01**	*p* = 0.10	*p* = 0.13	***p*** ** = 0.04**	*p* = 0.001	***p*** ** = 0.02**	***p*** ** = 0.001**	*p* = 0.11	*p* = 0.79	-

Abbreviation: ρ = correlation coefficient. Significant correlations are marked as * *p* < 0.05 and ** *p* < 0.01.

**Table 7:** Proportions of HOMA-IR categories.

Distribution of HOMA-IR Categories	Patients, n (%)
<1.0	10 (5.6%)
1.0–1.99	42 (23.4%)
2.0–2.99	36 (20.2%)
≥3.0	90 (50.8%)

**Table 8:** Comparison of incidence/prevalence and risk factors of PMOS.

Parameter	Current Study	GCC (4 Countries)	KSA	Global
**PMOS Prevalence**	Focused cohort	21.5–35.5%	18–56%	6–15%
**Mean Age**	31.9 ± 5.3	30–34	28–33	27–32
**Obesity**	43.8%	30–50%	35–60%	20–40%
**Primary Infertility**	61.8%	50–65%	55–60%	40–55%
**Secondary Infertility**	38.2%	35–50%	40–45%	45–60%
**HOMA-IR**	High	High	High	Variable
**Hyperandrogenism**	Present	Common	Common	Less severe
**BMI-Infertility**	Strong	Strong	Strong	Moderate
**Metabolic Syndrome**	High	High	High	Moderate

## Data Availability

All data were presented in the manuscript and can be obtained from the PI on a reasonable request and signed data sharing agreement with the military hospital.

## Abbreviations

PCOS	Polycystic Ovary Syndrome
LH/FSH	Luteinizing Hormone/Follicle-Stimulating Hormone
BMI	Body Mass Index
PMOS	Polyendocrine Metabolic Ovarian Syndrome
